# All-Optical 1-to-8 Wavelength Multicasting at 20 Gbit/s Exploiting Self-Phase Modulation in Dispersion Flattened Highly Nonlinear Photonic Crystal Fiber

**DOI:** 10.1155/2014/828179

**Published:** 2014-02-24

**Authors:** Zhan-Qiang Hui

**Affiliations:** Xi'an University of Posts and Telecommunications, Xi'an 710121, China

## Abstract

All-optical multicasting of performing data routing from single node to multiple destinations in the optical domain is promising for next generation ultrahigh-peed photonic networks. Based on the self-phase modulation in dispersion flattened highly nonlinear photonic crystal fiber and followed spectral filtering, simultaneous 1-to-8 all-optical wavelength multicasting return-to-zero (RZ) signal at 20 Gbit/s with 100 GHz channel spaced is achieved. Wavelength tunable range and dynamic characteristic of proposed wavelength multicasting scheme is further investigated. The results show our designed scheme achieve operation wavelength range of 25 nm, OSNR of 32.01 dB and *Q* factor of 12.8. Moreover, the scheme has simple structure as well as high tolerance to signal power fluctuation.

## 1. Introduction 

All-optical wavelength multicasting is an important function for increasing the efficiency and flexibility of wavelength-division-multiplexing/optical time division multiplexing (WDM/OTDM) optical networks, which involves transmitting a message from a source to multiple destinations simultaneously and has become the subject of intensive studies [1, 2]. Many bandwidth-intensity services in metro and access networks such as IP-TV, video distribution, and teleconferencing require reliable high-speed multicasting since it provides many benefits, especially simplification of network layer protocols and optical network design [3, 4].

So far, a variety of all-optical multicasting technologies has been demonstrated, such as using cross-gain modulation (XGM) [5], cross-phase modulation (XPM) [6], four-wave mixing (FWM) [7], and nonlinear polarization rotation (NPR) [8] in semiconductor optical amplifiers (SOAs), cross-absorption modulation (XAM) [9] in electro-absorption modulators (EAMs), cascaded sum- and difference-frequency generation in a periodically poled lithium niobate (PPLN) waveguide [10, 11], FWM in conventional dispersion-shifted highly nonlinear optical fibers (HNLFs) [12–14] or dispersion-flattened highly nonlinear photonic crystal fiber (DF-HNL-PCF) [15], and transient cross-phase modulation in DF-HNL-PCF [16]. Nevertheless, all these schemes have their respective drawbacks. For example, relatively long gain recovery time in SOAs [5] ultimately limits their operation speed, and a high manufacturing cost may prevent PPLN waveguides from practical applications; while a walk-off between short pulses due to dispersion in traditional HNLFs [13] can ultimately restrict the operation speed of such fiber-based signal processors. PCF based all-optical multicasting scheme [16, 17] exhibits obvious superiority due to its wide operation wavelength range and high nonlinearity, as well as flexible and controllable dispersion characteristics [18]. However, the operation speed of most of the PCF based all-optical multicasting schemes is at 10 Gbit/s until now, but future all-optical networks will be needed to support ultrahigh-speed communications. In addition, all these reported works mainly deal with the operation principle of wavelength multicasting using PCF cascaded with optical band-pass filter (OBPF), and the system is carried out only for the fixed signal wavelength. From the viewpoint of engineering applications, the operation wavelength range is an important issue in the design of wavelength multicasting schemes, which can affect the capacity of WDM/OTDM systems and the performance of wavelength switching/routing networks. Another thing we should not neglect is that some unexpected variety including input signal powers will affect the wavelength multicasting signal quality under practical networks environment. However, no attentions are paid to these topics, which are very important to the feasibility of the all-optical wavelength multicasting scheme.

In this paper, we demonstrate a simple approach to achieve all-optical one-to-eight channel wavelength multicasting at 20 Gbit/s in a 100 m DF-HNL-PCF. The input return-to-zero (RZ) data signal with high peak power is injected into the DF-HNL-PCF, which induces a varying refractive index of the medium due to the optical Kerr effect and further causes an intensity-dependent phase shift in the pulse, generating spectral broadening. When different spectral component is selected by using a central wavelength tunable OBPF simultaneously as output channel, the all-optical one-to-eight wavelength multicasting is achieved. We also carry out an experiment investigation into the wavelength tuning ranges of the resulting wavelength multicasting scheme. Moreover, the influences of the input signal power on the multicasting signal quality are exploited. The proposed wavelength multicasting scheme has advantages of simple configuration, wide wavelength tunable range, efficient operation with high tolerance to power fluctuation, and ultrafast response.

## 2. Experimental Setup

**Figure 1:**
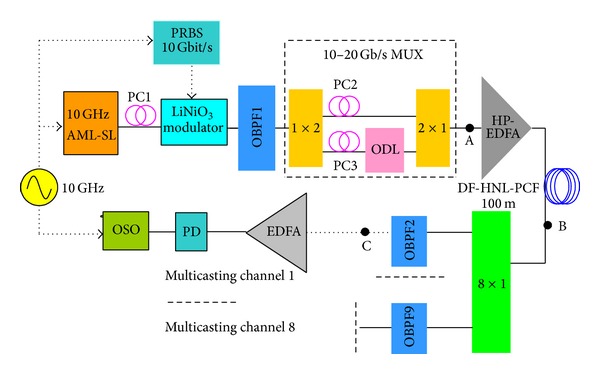
Experimental setup of DF-HNL-PCF based all-optical wavelength multicasting scheme.

The experimental setup for our all-optical wavelength multicasting scheme is illustrated in Figure 1, which basically consists of a high power erbium doped fiber amplifier (HP-EDFA), a 100 m DF-HNL-PCF, and an OBPF. The wavelength multicasting scheme uses the DF-HNL-PCF as nonlinear medium and the OBPF as spectral filtering device. In our experiment, the pulses with wavelength at *λ*_signal_ = 1555.2 nm from an actively mode-locked semiconductor laser are modulated by a LiNbO_3_ modulator at 10 Gbit/s 2^31^-1 bits pseudorandom binary sequences (PRBS) with a polarization controller (PC) at its input to align the state of polarization of the pulse train with the transmission axis of the modulator and then fed into a fiber-based interleaver that performs optical time division multiplexing to produce a 20 Gbit/s optical pulse signal for using in the experiments of all-optical wavelength multicasting. The PC2 and PC3 are used in MUX to adjust the polarization state of the two channels of the 20 Gbit/s OTDM signal. After passing through a HP-EDFA (Keopsys: KPS-CUS-BT-C-35) for power amplification, the 20 Gbit/s RZ signals are then launched into the 100 m DF-HNL-PCF (prepared by Crystal Fiber A/S). The RZ signal achieves sufficient spectral broadening induced by self-phase modulation (SPM) effect when passing through the DF-HNL-PCF. The nonlinear coefficient of the DF-HNL-PCF is 11 W^−1 ^km^−1^ and its dispersion is −0.5 ps/(nm*·*km) at 1550 nm. It has flattened dispersion slope of less than 0.01 ps/nm^2^ *·*km over 1500–1600 nm. The all-optical wavelength multicasting is demonstrated by exploiting an OBPF with tunable central wavelength to slice the broadened spectrum into eight channels with 100 GHz channel separation following the ITU grid.

## 3. Numerical Simulations

To explain the intrinsic mechanism of our all-optical wavelength multicasting scheme, a potent DF-HNL-PCF model should be developed to predict the DF-HNL-PCF operation. Ultrashort RZ pulse propagation in DF-HNL-PCF is a complex process which is involved in various nonlinear processes such as SPM, self-steepening, and stimulated Raman scattering (SRS) [19]. Without considering the polarization coupling, a generalized scalar nonlinear Schrödinger equation can be used to describe the ultra-short pulse propagation inside the DF-HNL-PCF [20]:

(1) ∂A∂z=∑m≥2im+1βmm!∂mA∂τm−αint⁡2A+iγ(1+iω0∂∂τ) ×[A(z,τ)]∫−∞τdτ′R(τ−τ′)|A(z,τ′)|2,

where *A* = *A*(*z*, *t*) is the electric field amplitude, *z* is the longitudinal coordinate along the fiber, *τ* is the time in a reference frame traveling with the pump light, *β*_*m*_ is the *m*th-order dispersion coefficient at the central frequency *ω*_0_, *α*_int⁡_ is fiber loss, *γ* = *n*_2_ *ω*_0_/(*cA*_eff_) is the nonlinear coefficient, *n*_2_ = 2.0 × 10^−20^ m^2^/W is the nonlinear refractive index of fused-silica glass, and *A*_eff_ is the effective mode area of the fiber.

The first and second terms on the right-hand side (RHS) of (1) represent the *m*th-order dispersion and loss, respectively. The third term includes SPM, self-steepening, optical shock formation, and intrapulse Raman scattering. The response function *R*(*τ*) = (1 − *f*_*R*_)*δ*(*τ*) + *f*_*R*_ *h*_*R*_(*τ*) includes both instantaneous electronic and delayed Raman contributions, with *f*_*R*_ = 0.18 representing the contribution of Raman response to the instantaneous nonlinear polarization, and *h*_*R*_(*τ*) = (*τ*_1_^2^ + *τ*_2_^2^)/(*τ*_1_ *τ*_2_^2^)exp⁡⁡(−*τ*/*τ*_2_)sin⁡(−*τ*/*τ*_1_) is Raman response function of silica fiber, where *τ*_1_ = 12.2 fs and *τ*_2_ = 32 fs.

**Figure 2:**
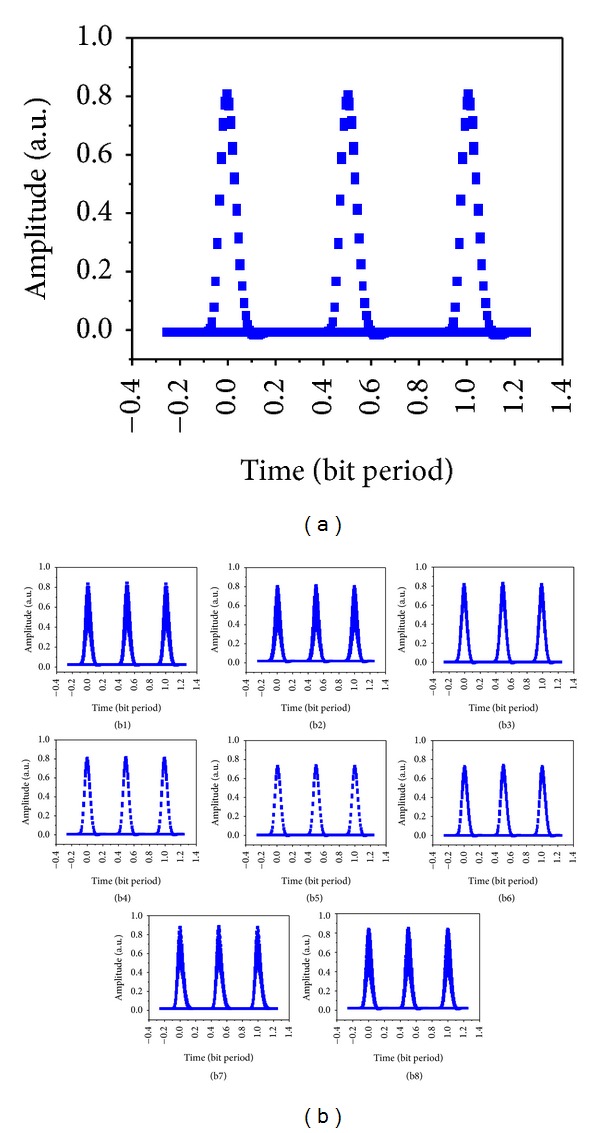
Eye diagrams of the simulation for (a) original RZ signal and (b1)–(b8) eight filtered multicasting channels.

Equation (1) can be numerically solved by using the split-step Fourier method. The fiber parameters in our simulation agree well with that of practical 100 m DF-HNL-PCF used in our experiment. The DF-HNL-PCF has pure fused silica strand of diameter 2.1 *μ*m surrounded by air. The nonlinear coefficient of the HNL-PCF is 11 W^−1 ^km^−1^ and its dispersion is −0.5 ps/(nm km) at 1550 nm. The optical loss is *α*_int⁡_ = 9 dB/km. The OBPF used in the system is modeled as a Gaussian filter with full width half maximum (FWHM) of *B*_0_. The transfer function of the filter is

(2) $$\begin{array}{c} F\left(\omega \right)=\exp\left[-2\ln2•{\left(\frac{\omega -\omega_{f}}{B_{0}}\right)}^{2}\right], \end{array}$$

where *ω*_*f*_ is the central angle frequency of the OBPF. The filter is implemented in the frequency domain using a fast Fourier transform (FFT) algorithm. The input pulses are assumed to have the form

(3) $$\begin{array}{c} A\left(0,\tau \right)=\sqrt{P_{0}}\text{sech}\left(\frac{\tau }{T_{0}}\right), \end{array}$$

where *P*_0_ is peak power with central wavelength at 1555.2 nm. *T*_0_ is related to the FWHM by *T*_FWHM_ = 1.763*T*_0_. In our simulation, the parameters are as follows: *B*_0_ = 50 GHz, *P*_0_ = 28 dBm, *T*_0_ = 5 ps, the responsivity of photodetector PIN is 1 A/W, and the dark current is 10 nA. The simulation results are shown in Figure 2. The original RZ signal is modulated by a Mach-Zehnder modulator at 10 Gbit/s 2^31^-1 bits PRBS and then fed into a fiber-based interleaver that performs optical time division multiplexing to produce a 20 Gbit/s optical signal for wavelength multicasting, whose eye diagram is shown in Figure 2(a). In contrast, the eye diagrams of eight multicasting channels are also given in Figure 2(b).

## 4. Results and Analysis

**Figure 3:**
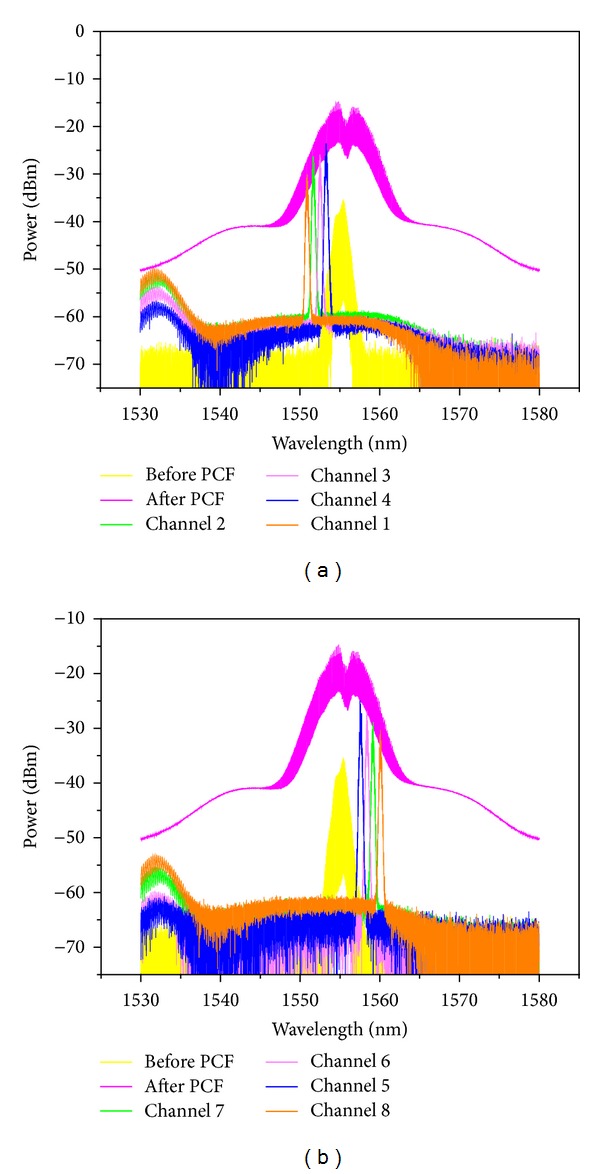
Optical spectrum measured at the input, the output of PCF, and the output of OBPF for (a) left sideband and (b) right sideband.

**Figure 4:**
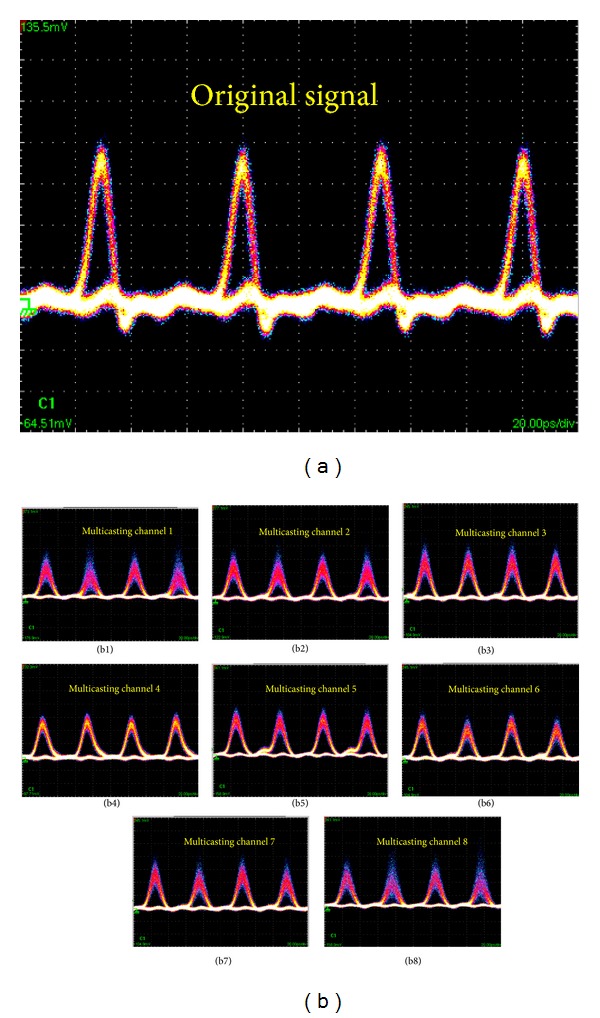
Eye diagrams of (a) the original input RZ signal and (b) the eight filtered multicasting channel signals. Time base: 20 ps/div.

The experimental results are shown in Figures 3 and 4.  Figure 3 shows the optical spectra obtained at the input of the PCF, output of the PCF, and after OBPF (corresponding to points A, B, and C, respectively, shown in Figure 1). The input data signal is 20 GHz at 1555.2 nm with a FWHM about *τ*_FWHM_ = 1.9 ps. The input and output power of HP-EDFA is 1.2 and 24.3 dBm. The output spectrum from the PCF is broadened obviously due to SPM induced by optical pulse with high peak power. The signal power is reallocated across the broadened optical spectrum. The 0.38 nm bandwidth OBPF followed with tunable central wavelength from 1530 nm to 1570 nm is used to filter the broadened spectrum component. Figures 3(a) and 3(b) are for filtering the left and right sideband with 100 GHz channel space, respectively. Note the central component of the broadened spectrum is unsuitable for filtering as multicasting signal due to low RZ signal quality, which can be used for format conversion [21]. Then, all-optical one-to-eight channel wavelength multicasting is obtained. The eight channels are denoted as channel 1 to 8 from the left- to right-most channel accordingly. The eye diagrams of the original RZ signal and the eight output multicasting channels are depicted in Figure 4. Generally, clearly and widely open eye diagrams of the multicasting signals are obtained for all eight channels with good output signal quality. But the signal quality is slightly decreased for all eight channels from central to sideband symmetrically. The multicasting signals of channel 4 and channel 5 have better *Q* factor than others due to higher optical signal-to-noise ratio (OSNR) of them. On the contrary, some evident peak-to-peak jitter and amplitude fluctuations are observed in channel 1 and channel 8. This can be attributed to the relatively low output OSNR as a result of insufficient phase modulation for sideband spectrum. Note that because of lack of high quality Bit-error-rate test equipment in our laboratory, the performance test like Bit-error-rate is beyond the scope of this paper and will be considered in our future work.

### 4.1. Wavelength Tunability

**Figure 5:**
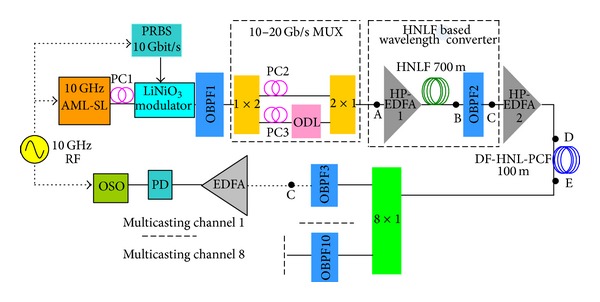
Experimental setup of DF-HNL-PCF based all-optical wavelength multicasting scheme.

**Figure 6:**
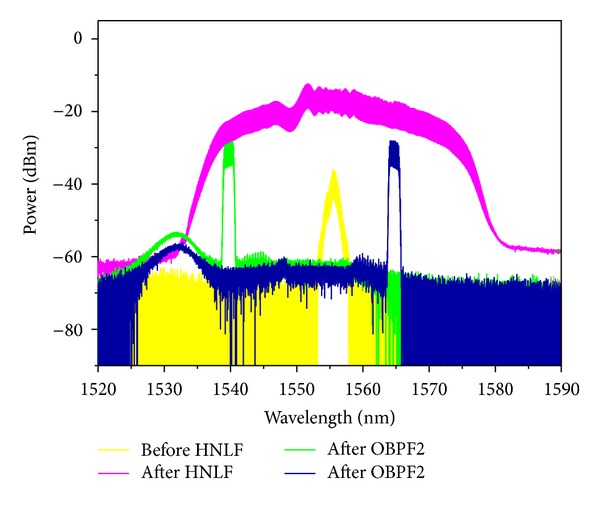
Optical spectrum measured at the input, the output of 700 m HNLF, and the output of OBPF2.

**Figure 7:**
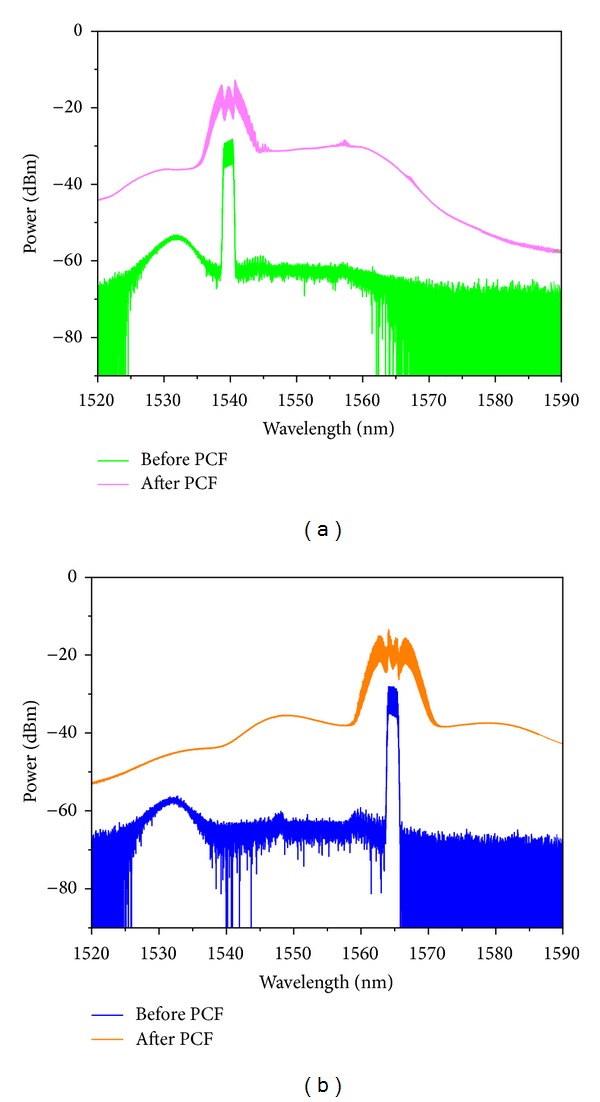
Optical spectrum measured at the input and the output of 100 m DF-HNL-PCF for (a) *λ*_signal_ = 1540 nm and (b) *λ*_signal_ = 1565 nm.

**Figure 8:**
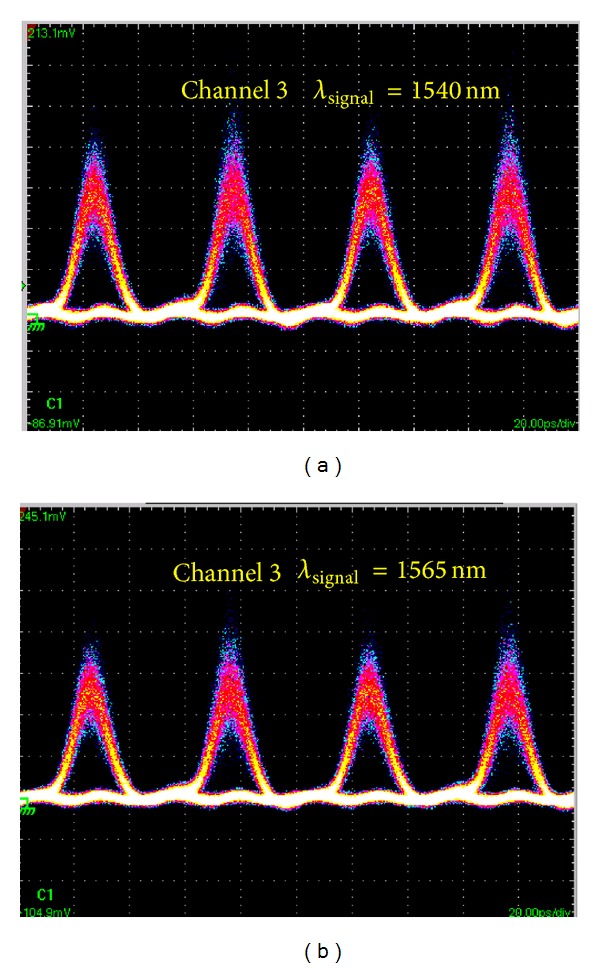
Eye diagrams of the filtered multicasting signals of channel 3 for (a) *λ*_signal_ = 1540 nm and (b) *λ*_signal_ = 1565 nm. Time base: 20 ps/div.

All-optical wavelength multicasting scheme with wide wavelength operation range is highly desirable in next generation ultrahigh-speed photonic networks. The nonlinear PCF exhibits weak group velocity dispersion (GVD) and a small dispersion slope, which provides the feasibility of broadband operating. Hence, we carry out a systematic investigation into the wavelength tuning range of resulting wavelength multicasting scheme. In our test, the RZ signal with different wavelength is obtained by a HNLF based wavelength converter, which consists of a HP-EDFA (Amonics: AEDFA-33-B-FA), a 700 m HNLF, and a 1.5 nm bandwidth OBPF (Santec: OTF-950). The experimental setup is shown in Figure 5. The nonlinear coefficient of the HNLF is 9 W^−1^·km^−1^ and its dispersion is −2.42 ps/(nm·km) at 1550 nm with dispersion slope of 0.02 ps/nm^2^·km. Its internal loss is less than 0.43 dB/km. Due to high nonlinear coefficient of HNLF, sufficient super continuum spectrum broadening of 42 nm is observed. We filter out different components of broadened spectrum and then obtained RZ signal at different wavelengths. To demonstrate the wide operation wavelength range, the central wavelength of OBPF is set at 1540 and 1565 nm, respectively. The spectrum at the input, output of the HNLF, and after OBPF2 (corresponding to A, B, and C in Figure 5) is shown in Figure 6. Furthermore, the wavelength converted 20 Gbit/s RZ signal is injected into 100 m DF-HNL-PCF after power amplification for one-to-eight wavelength multicasting. The spectrum at the input, output of the DF-HNL-PCF (corresponding to D and E in Figure 5) is shown in Figures 7(a) and 7(b) for *λ*_signal_ = 1540 nm and *λ*_signal_ = 1565 nm, respectively. Obviously, spectrum broadening induced by SPM is observed. Different sideband components are filtered and serve as different multicasting channels, and subsequent eye diagrams monitoring shows clear eye diagrams are obtained. We just show one typical result of wavelength multicasting at channel 3 for *λ*_cw_ = 1540 nm and *λ*_cw_ = 1565 nm (Figure 8), though we can get converted signal at any of 8 channels continuously. The results demonstrate that our proposed all-optical wavelength multicasting scheme can achieve a wide wavelength multicasting range of nearly 25 nm, which is substantially limited by the operation wavelength range of wavelength converter.

### 4.2. Impact of Signal Power

**Figure 9:**
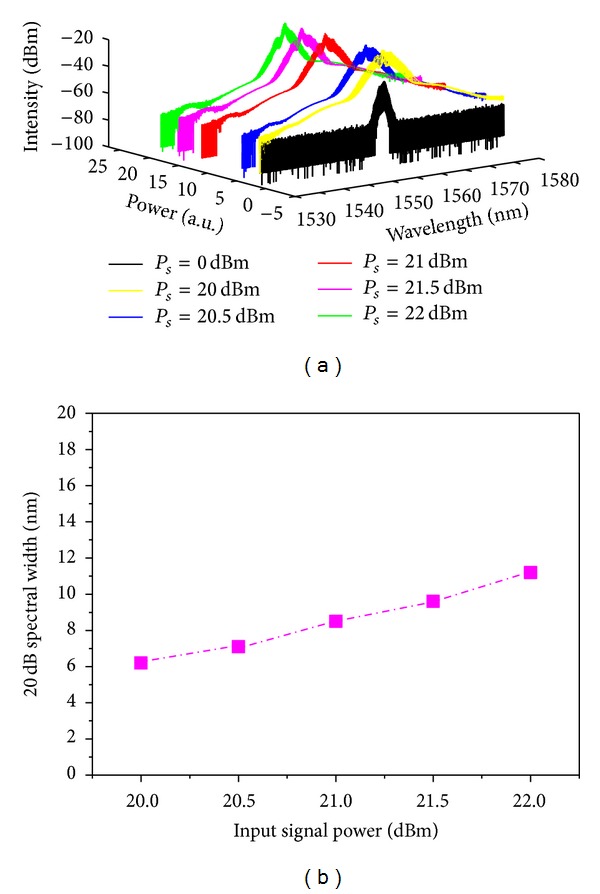
Spectrum characteristics of DF-HNL-PCF based wavelength multicasting scheme: (a) the SPM broadened spectrum and (b) 20 dB spectrum bandwidth versus input signal power.

In practical optical fiber communication networks, the power fluctuations happen occasionally, which will lower the performance of optical networks. From the viewpoint of engineering design and applications, the tolerance to changes of signal power is an important issue on the design of all-optical wavelength multicasting scheme. Then, to facilitate the practical design, we carry out an investigation into the impact of signal power on the *Q* factor and optical signal-to-noise ratio (OSNR) of multicasting signals. In our experiment, the signal wavelength is fixed at 1555.2 nm. The OBPF bandwidth is maintained at 0.38 nm as before. The results are shown in Figure 9.  Figure 9(a) shows the broadened spectra under different input signal power. The signal power varies from 20 dBm to 22 dBm in step of 0.5 dB in our test. The original signal spectrum is also given for comparison. The 20 dB spectral width at different input signal power is measured and shown in Figure 9(b). It is clear that 20 dB spectral widths are broadened obviously, which is proportional to the input signal power.

**Figure 10:**
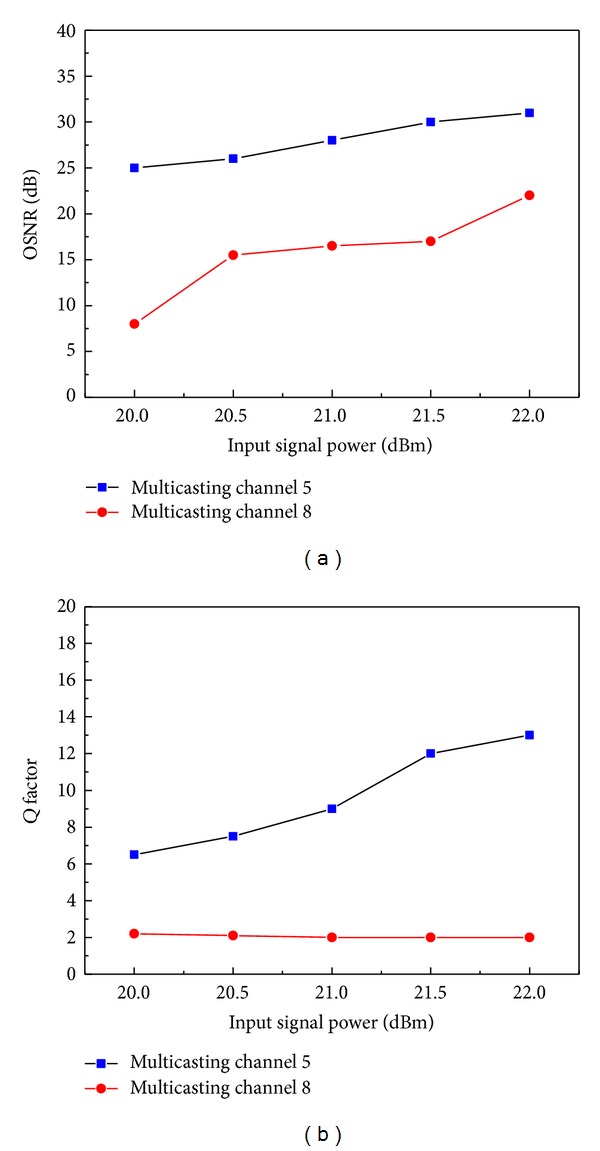
Dynamics characteristics of DF-HNL-PCF based wavelength multicasting scheme: (a) the OSNR and (b) *Q* factor versus input signal power.

*Q* factor and OSNR are key parameters which are always used to evaluate the signal quality. The relationship of the *Q* factor versus input signal power and OSNR versus input signal power for such a DF-HNL-PCF based wavelength multicasting scheme is shown in Figures 10(a) and 10(b). We just show typical results of multicasting channel 5 and channel 8 as an example, though we can get results for all eight multicasting channels.  Figure 10(a) shows the OSNR of channel 8 increases more rapidly than channel 5, which can be attributed to the fact that the optical spectrum has wider sideband under stronger input power, while the *Q* factor of channel 8 increases slightly with the increase of input signal power in Figure 10(b). This means even the signal power decreases by 3 dB, the multicasting channel signal quality does not decrease obviously. The results show that the proposed all-optical multicasting scheme has some tolerance to the fluctuation of input signal power. This property is very useful for the engineering design and application of DF-HNL-PCF based all-optical wavelength multicasting scheme, because it can alleviate the strict requirement of controlling the light power in practical networks.

## 5. Conclusion

DF-HNL-PCF is capable of producing substantial spectral broadening due to SPM. The phenomenon can be used to achieve all-optical wavelength multicasting for using in the WDM/OTDM and package switching networks. All-optical one-to-eight wavelength multicasting of 20 Gbit/s RZ signal has been demonstrated using SPM in a 100 m DF-HNL-PCF. Eight multicasting channels are obtained with an adjacent channel spacing of 100 GHz by adjusting the central wavelength of OBPF. The operating wavelength range and dynamic characteristics of proposed multicasting scheme have been investigated. The results show that a broadband of 25 nm wavelength tuning range is obtained and the scheme has high tolerance to input signal power fluctuation. Our proposed scheme has advantages as follows: (1) simple implementation without any external light source, (2) easy to reach high *Q* factor, (3) high tolerance to signal power fluctuation, and (4) ultrafast response.

## References

[1] Gong L, Zhou X, Liu X (2013). Efficient resource allocation for all-optical multicasting over spectrum-sliced elastic optical networks. *Optical Communications and Networking*.

[2] Zhang F, Zhong WD, Xu Z, Cheng TH, Michie C, Andonovic I (2011). A broadcast/multicast-capable carrier-reuse WDM-PON. *Journal of Lightwave Technology*.

[3] Lim W, Yang Y, Milosavljevic M (2012). Multicast polling for 10G-EPON. *Electronics Letters*.

[4] Wang Y, Yu C, Luo T, Yan L, Pan Z, Willner AE (2005). Tunable all-optical wavelength conversion and wavelength multicasting using orthogonally polarized fiber FWM. *Journal of Lightwave Technology*.

[5] Contestabile G, Calabretta N, Proietti R, Ciaramella E (2006). Double-stage cross-gain modulation in SOAs: an effective technique for WDM multicasting. *IEEE Photonics Technology Letters*.

[6] Yan N, Silveira T, Teixeira A (2007). 40 Gbit/s wavelength multicast via SOA-MZI and applications. *Electronics Letters*.

[7] Contestabile G, Presi M, Ciaramella E (2004). Multiple wavelength conversion for WDM multicasting by FWM in an SOA. *IEEE Photonics Technology Letters*.

[8] Contestabile G, Calabretta N, Presi M, Ciaramella E (2005). Single and multicast wavelength conversion at 40 Gb/s by means of fast nonlinear polarization switching in an SOA. *IEEE Photonics Technology Letters*.

[9] Chow KK, Shu C (2003). All-optical wavelength conversion with multicasting at 6 × 10 Gbit/s using electroabsorption modulator. *Electronics Letters*.

[10] Furukawa H, Nirmalathas A, Wada N, Shinada S, Tsuboya H, Miyazaki T (2007). Tunable all-optical wavelength conversion of 160-Gb/s RZ optical signals by cascaded SFG-DFG generation in PPLN waveguide. *IEEE Photonics Technology Letters*.

[11] Wang J, Sun JQ, Luo CH, Sun QZ, Zhang XL, Huang DX (2006). Single-to-multiple channel wavelength conversions and tuning of picosecond pulses in quasi-phase-matched waveguides. *Chinese Physics Letters*.

[12] Ma J, Yu J, Yu C (2006). Wavelength conversion based on four-wave mixing in high-nonlinear dispersion shifted fiber using a dual-pump configuration. *Journal of Lightwave Technology*.

[13] Devgan P, Tang R, Grigoryan VS, Kumar P (2006). Highly efficient multichannel wavelength conversion of DPSK signals. *Journal of Lightwave Technology*.

[14] Krcmarik D, Karasek M, Radil J, Vojtech J (2007). Multi-wavelength conversion at 10 Gb/s using cross-phase modulation in highly nonlinear fiber. *Optics Communications*.

[15] Fok MP, Shu C (2007). Multipump four-wave mixing in a photonic crystal fiber for 6 × 10 Gb/s wavelength multicasting of DPSK signals. *IEEE Photonics Technology Letters*.

[16] Hui ZQ, Zhang JG (2011). All-optical wavelength multicasting exploiting cross-phase modulation in a dispersion-flattened nonlinear photonic crystal fiber. *Laser Physics*.

[17] Kwok CH, Lee SH, Chow KK, Shu C, Lin C, Bjarklev A Polarization-insensitive all-optical wavelength multicasting by self-phase-modulation in a photonic-crystal fiber.

[18] Russell PSJ (2006). Photonic-crystal fibers. *Journal of Lightwave Technology*.

[19] Agrawal GP (2001). *Nonlinear Fiber Optics*.

[20] Zhu Z, Brown TG (2004). Effect of frequency chirping on supercontinuum generation in photonic crystal fibers. *Optics Express*.

[21] Hui ZQ, Gong JM, Liang M, Zhanga MZ, Wub HM (2013). Demonstration of all-optical RZ-to-NRZ format conversion based on SPM in a dispersion flattened highly nonlinear photonic crystal fiber. *Optical and Laser Technology*.

